# Hard work, long hours, and Singaporean young adults' health—A qualitative study

**DOI:** 10.3389/fpubh.2023.1082581

**Published:** 2023-06-12

**Authors:** Jodie Leu, Salome A. Rebello, Ginny M. Sargent, Matthew Kelly, Cathy Banwell

**Affiliations:** ^1^National Centre for Epidemiology and Population Health, The Australian National University, Canberra, ACT, Australia; ^2^Saw Swee Hock School of Public Health, National University of Singapore, Singapore, Singapore

**Keywords:** work time, health practices, diet, physical activity, obesity, health promotion, preventing non-communicable diseases, burden of disease

## Abstract

**Background:**

As young adults in their 20s to 30s transitioning toward new careers and independence, their dietary and physical activity practices often change, increasing their risk of weight gain. This study explored the ways that Singaporean young adults perceived and experienced the interaction between their working hours, work, and health practices.

**Methods:**

This research used semi-structured interviews to explore the perspectives and experiences of participants. Purposive and snowball sampling was used to recruit 15 men and 18 women, aged 23 to 36, who had worked full-time at their respective jobs in Singapore for at least 1 year. An inductive and deductive thematic analysis approach was employed.

**Results:**

Young working adults' commitment to work was driven by a hard-working culture, a desire to attain better jobs and remuneration, and to fulfill cultural expectations to support their multi-generation families. Their non-work time was largely spent recuperating from work by socializing over food and in sedentary activities.

**Conclusion:**

For young working adults, long work hours are normalized, even though they are a barrier to healthy diets and physical activity. Existing social and institutional norms support a culture that values commitment to work and encourages young adults to devote long hours to building a sound financial future and achieving personal and cultural aspirations. These findings have implications for long-term population health and should be considered in health promotion activities targeting young adults and barriers.

## Introduction

There are strong indications that key risk factors of preventable non-communicable diseases are increasing in Singapore's population: insufficient exercise and obesity are increasing with the consumption of more calorie-dense and nutrient-poor food (1–3). Poor diets and little physical activity are within the top 10 risk factors of premature death and disability in Singapore (4). Furthermore, preventable non-communicable diseases are experienced at younger ages. The country has the highest proportion of younger diabetes patients in Asia (5), while young adults, aged 30–39 years, have the highest prevalence of obesity at 12.4%, of any age group in Singapore (6).

Singapore is a multi-ethnic South-East Asian country where the majority are Chinese (74.3%), followed by Malay (13.4%), Indian (9.0%), and other ethnicities (3.2%) (7). Singapore's British colonial heritage is overlaid with a distinctive multi-cultural, modern society that reflects the East Asian background of its' largest ethnic population. It is famous for its diverse dining offerings ranging from outdoor hawker centers to expensive restaurants (8). Though small, Singapore has become a high-income, urban nation, since independence in 1965. At 44.6 h per week, its' workforce has among the longest average working hours in the world (9). Almost 90% of Singaporean young adults aged 25–34 are employed, and they work an average of 44 hours per week like their older counterparts (10).

Working more than 40 h per week diminishes workers' ability and time for self-care, negatively impacting their physical and mental health and contributing to obesity and related chronic health conditions in Australia (11–13) and the USA (14). Other international studies have found that stress from personal and work lives can affect dietary practices and have negative consequences for body weight and health (15–18).

Long working hours are normative in Singapore and are part of a broader cultural acceptance of work as a key element of Singaporean culture (19). Young working adults seem vulnerable to weight gain; as they leave home, settle into working life, and start a new family, their dietary and physical activity practices change (20). After starting work, 60% of Singaporean workers gain weight, putting on an average of 3 kg per year for the first 8 years with 17% gaining more than 20 kg after job commencement (21). Younger people are particularly dissatisfied with their weight with 30% of under 25 year olds restricting their diet (22). Their diets are not rated as particularly healthy with most Singaporean university students not meeting recommended daily servings of fruit and vegetables (23). Additionally, while about one-third (33.4%) of adults regularly took part in leisure time physical activity, 42.9% did not exercise at all (24).

Even though Singapore is conspicuous for its long work hours, the topic has not been investigated in depth. This study explores young adults' work hours in the context of work culture, encompassing shared values, beliefs, attitudes, behavior, and customs toward work (25). Understanding how young adults perceive and respond to the impact of long working hours and other work-related normative practices in their daily lives and how these aspects constrain their health practices is important for two reasons. First, it is likely that people's perceptions of time scarcity influences the amount of time they think they have for discretionary activities or those that are not committed to work, domestic chores, and personal care (26). Second, it is more effective to prevent weight gain than to lose weight (27). An in-depth awareness may guide intervention efforts to reduce barriers to health practices and slow the development of chronic health conditions in later life.

This study focuses on young people working in sedentary white-collar jobs who are most likely to be at risk of weight gain. It seeks to understand their perspectives and experiences on how their work hours and work culture influence their health practices. Even though Singapore is renowned for its strong work ethic (19), young workers' perceptions of the impact of their work environment on health remain underexplored.

## Methods

### Study design

Increasingly, it is recognized that health knowledge alone is not enough to change health practices that reflect subjective experiences embedded in a socio-environmental context. As such, an understanding of nuanced, inter-related, and multi-level relationships is useful. Consequently, a qualitative approach is suited to explore these relationships. This study was comprised of in-depth, semi-structured interviews supported by participant information and standardized questionnaires. It is part of a year-long study using a focused ethnographic approach (28) to explore a social phenomenon and provide insights into the interactions between the everyday working lives of Singaporean young adults and their dietary and health practices.

#### Recruitment

**Figure 1:**
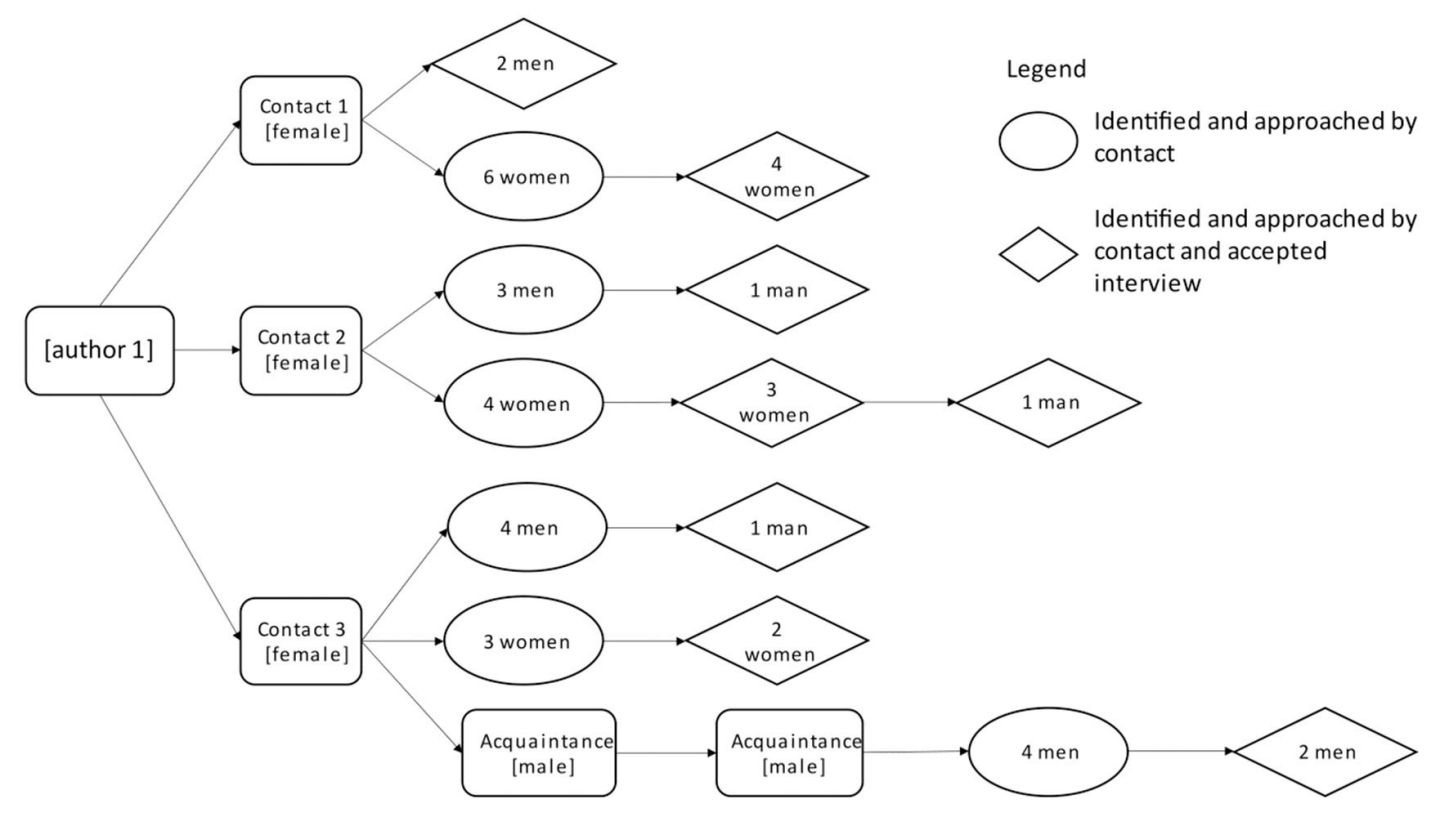
Visual representation of the arms-length recruitment of 16 participants who were acquaintances of contacts. None of the contacts were interviewed.

Eligible participants were young adults aged 21 to 36 living in Singapore (Singaporean citizens or permanent residents who had lived in Singapore for at least 1 year) and working full-time (at least 38 h per week for at least 1 year at the same job). Purposive and snowball samplings were used to recruit participants of similar ages and life experiences who could provide insight into the phenomena studied (29). To mitigate potential recruitment bias, participants were recruited in either one of the two ways: (1) 100 men and 100 women from a list of previous research participants in studies conducted by the National University of Singapore (NUS) who had stated they were willing to be contacted for future research purposes were called by the lead author and (2) a convenience sample was approached following expressed interest after an introduction by the lead author's contacts, using their network of acquaintances (Figure 1). After initial contact, interested potential participants were emailed a letter of invitation, with a study information sheet, a copy of the consent form, and a time-use diary. Prior to participation, all participants were fully informed regarding what the study would entail and their right to refuse to answer any questions and withdraw at any time. With their permission, a one-off interview was arranged. Written informed consent was obtained from all participants. No participants withdrew participation from the study. Upon the completion of research activities, participants were offered a supermarket voucher worth 20 Singapore Dollars (SGD)^1^ to thank them for their time.

Recruitment took place between June and October 2016 and continued until data saturation was reached, meaning that little additional novel data were emerging (30). A total of 33 participants were recruited: 17 participants (eight men and nine women) from previous NUS studies and 16 participants (seven men and nine women) from the lead author's contacts.

#### Data collection

An interview guide, developed from related work by Eckermann (31), Hubert (32), Mack and colleagues (33), and Bisogni and colleagues (34–38), was used (see Supplementary material 1). During face-to-face interviews, open-ended questions were deployed flexibly, to gain insight into participants' understandings and beliefs related to their perceptions of health; narratives about their food and dietary practices in the context of the foodscape; their work and time use; physical activity; and broader sociocultural influences such as family, friends, social media elements, and government initiatives.

Participants were also asked to complete short socio-demographic questionnaires that included the level of income and working hours. Standardized questionnaires were used to collect data on work satisfaction (39), time pressure (40), self-rated health (41), and self-reported height and weight to calculate body mass index (BMI) and physical activity [using the global physical activity questionnaire, GPAQ (42)]. A 3-day time-use diary (2 weekdays and 1 weekend day), adapted from the Australian Bureau of Statistics Time Use Survey (43), was also administered to record time spent on daily activities such as sleep, commuting, and working. Additionally, photos that were voluntarily provided by participants on the days they recorded their time-use diaries were used. These mixed methods helped provide a comprehensive understanding of the phenomena studied (44).

All data were collected by the lead author who was a female Asian postgraduate student of similar age to the participants, with prior experience of living in Singapore, and trained in qualitative methodologies. Interviews were conducted in English and audio recorded and noted. Most interviews, lasting about an hour, were conducted in public spaces at the convenience of the interviewee. The process of data collection and data saturation reached was discussed among all authors. A pseudonym was assigned or chosen by participants to protect their anonymity and maintain confidentiality. For a year, field notes focusing on food-related spaces, such as hawker centers, were taken.

Ethical approval was attained from the Australian National University (ANU) Human Research Ethics Committee (Protocol: 2015/813) and the NUS Institutional Review Board (B-16-076). Written informed consent was obtained from all participants. All the methods in this study were performed in accordance with the relevant guidelines and regulations.

### Analysis

**Figure 2:**
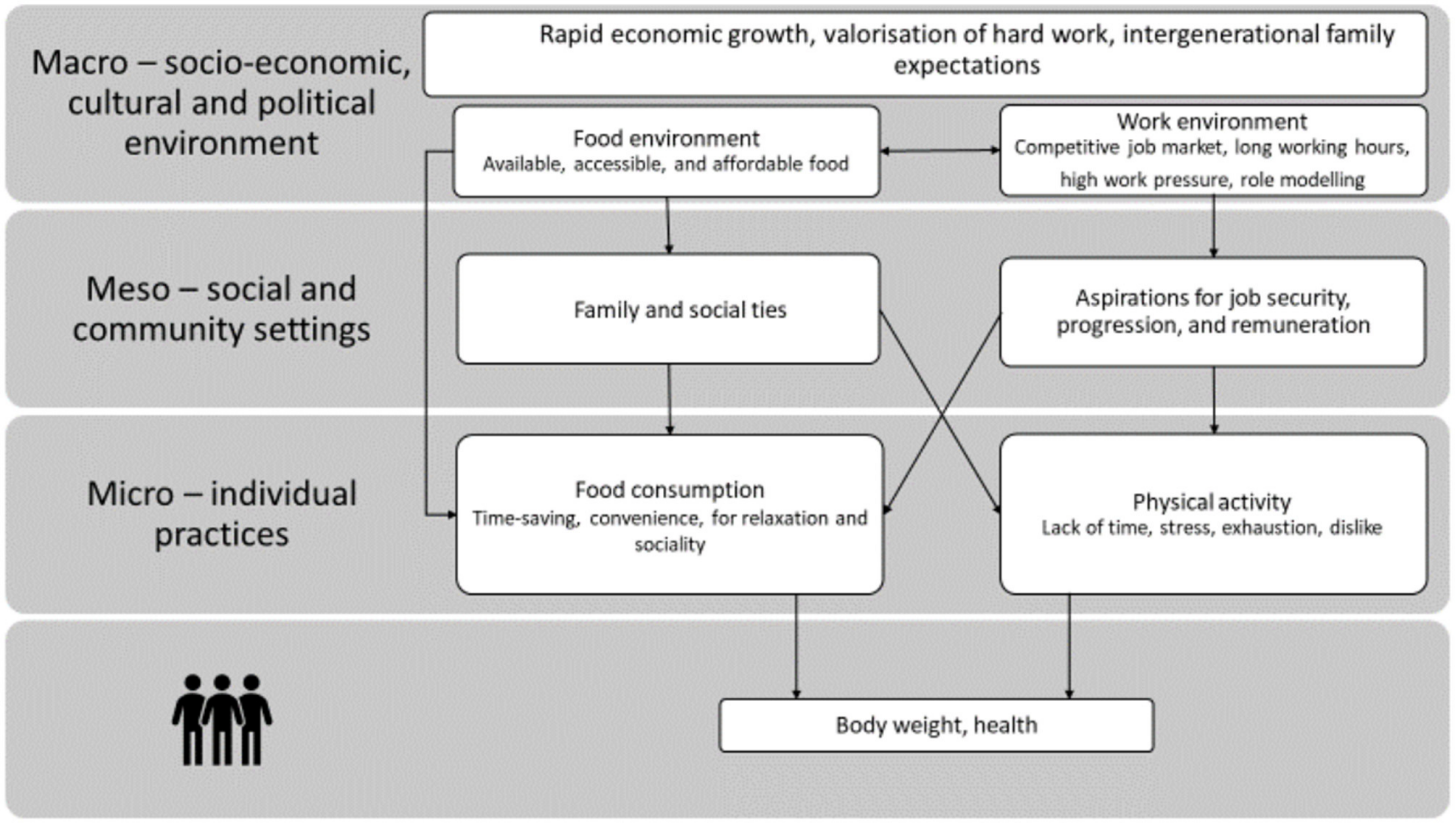
Conceptual map illustrating the multi-level aspects of the four main themes identified from analysis and how they influence participants' health and body weight: working hours and time use; social norms and long work hours; the influence of work on eating practices; and the influence of work on physical activities.

Interviews were transcribed and the lead author read and reread the transcripts for familiarization. Member-checking of transcripts was not undertaken to lessen participant burden. An inductive and deductive thematic analysis approach (45) was adopted for its flexibility. The deductive elements were informed by existing literature characteristic of a focused ethnographic approach and were linked with the main research questions. Our inductive thematic analysis sought to identify codes and then themes that were apparent in the data. Transcripts were coded in Atlas.ti 8 Windows (46), and key concepts and themes were discussed among all authors. Identified themes were further organized into individual, community, and environmental levels guided by a framework based on the social determinants of health which describe the interplay of individual and structural factors (47) (Figure 2).

Questionnaire data provided background demographic, work, and physical activity information. Ethnic, marital status, and educational levels were categorized according to the standard used in Singapore's population statistics, and occupations were defined by the Singapore Standards Occupational Classification 2015 (Version 2018) (48). Based on WHO definitions, participants' BMIs were categorized as underweight (BMI < 18 kg/m^2^), normal weight (BMI 18.5–24.9 kg/m^2^), overweight (25.0–29.9 kg/m2), and obese (BMI ≥ 30 kg/m^2^) (49). Distributions and averages of scales and indices were calculated for the work satisfaction scale, time pressure scale, and self-rated health (39–41). Metabolic equivalents (METs) were calculated following the GPAQ analysis guide to classify physical activity intensity as high, moderate, and low (42). Participants' time-use diaries were tabulated to show the average amount of time spent on each of the activities pre-defined in the diary. The Consolidated Criteria for Reporting Qualitative Studies (COREQ) checklist was followed for the preparation of this study (50).

## Results

Most (69.7%) of the 15 male and 18 female participants identified as ethnically Chinese (Table 1) which reflects the ethnic composition of Singapore; Chinese (74.3%), Malay (13.4%), Indian (9.0%), and other ethnicities (3.2%) (7). Participants were aged between 23 and 36.

**Table 1:** Sociodemographic information of participants.

	**Total (*N* = 33)**	**Men (*N* = 15)**	**Women (*N* = 18)**
***N*** **(%)**		
**Ethnicity**			
Chinese	23 (69.7)	11 (73.3)	12 (66.7)
Malay	6 (18.2)	1 (6.7)	5 (27.8)
Indian	3 (9.1)	3 (20.0)	0 (0.0)
Other	1 (3.0)	0 (0.0)	1 (5.6)
	Mean ± SD		
**Age (years)**	28.7 ± 0.7	29.1 ± 3.2	28.3 ± 4.6
**BMI** (**kg/m**^**2**^)	22.3 ± 3.8	24.6 ± 3.0	20.5 ± 3.6
	N (%)		
**BMI categories**			
Underweight < 18.5	4 (12.1)	0 (0.0)	4 (22.2)
Normal weight, 18.5–24.9	22 (66.7)	10 (66.7)	12 (66.7)
Overweight, 25.0–29.9	5 (15.2)	3 (20.0)	2 (11.1)
Obese ≥ 30	2 (6.1)	2 (13.3)	0 (0.0)
**Living arrangements**			
With family	28 (84.8)	12 (80.0)	16 (88.9)
With housemates	3 (9.1)	2 (13.3)	1 (5.6)
Alone	2 (6.1)	1 (6.7)	1 (5.6)
**Marital status**			
Never Married	23 (69.7)	10 (66.7)	13 (72.2)
Married	10 (30.3)	5 (33.3)	5 (27.8)
**Number of children**			
0	27 (81.8)	13 (86.7)	14 (77.8)
1	1 (3.0)	1 (6.7)	0 (0.0)
2	4 (12.1)	1 (6.7)	3 (16.7)
3	1 (3.0)	0 (0.0)	1 (5.6)
**Highest completed level of education**			
Secondary	1 (3.0)	0 (0.0)	1 (5.6)
Diploma	8 (24.2)	3 (20.0)	5 (27.8)
Bachelor's	20 (60.6)	9 (60.0)	11 (61.1)
Professional certificates	1 (3.0)	1 (6.7)	0 (0.0)
Master's	3 (9.1)	2 (13.3)	1 (5.6)
**Occupation**			
Legislators, senior officials and managers	2 (6.1)	0 (0.0)	2 (11.1)
Professionals	24 (72.7)	13 (86.7)	11 (61.1)
Associate professionals and technicians	1 (3.0)	1 (6.7)	0 (0.0)
Clerical support workers	5 (15.2)	1 (6.7)	4 (22.2)
Service and sales workers	1 (3.0)	0 (0.0)	1 (5.6)
**Monthly income inclusive of Central Provident Funds (SGD)**			
1,000–2,000	2 (6.1)	1 (6.7)	1 (5.6)
2,000–3,000	7 (21.2)	2 (13.3)	5 (27.8)
3,000–4,000	6 (18.2)	2 (13.3)	4 (22.2)
4,000–5,000	12 (36.4)	5 (33.3)	7 (38.9)
6,000–10,000	6 (18.2)	5(33.3)	1 (5.6)

Our analysis identified four major work-related themes operating at different levels (Figure 2). These themes illuminate the ways in which the Singaporean work culture, particularly long hours, influences the health practices of young working adults.

### *Weekend is the only time to like... just collapse*: working hours and time use

**Figure 3:**
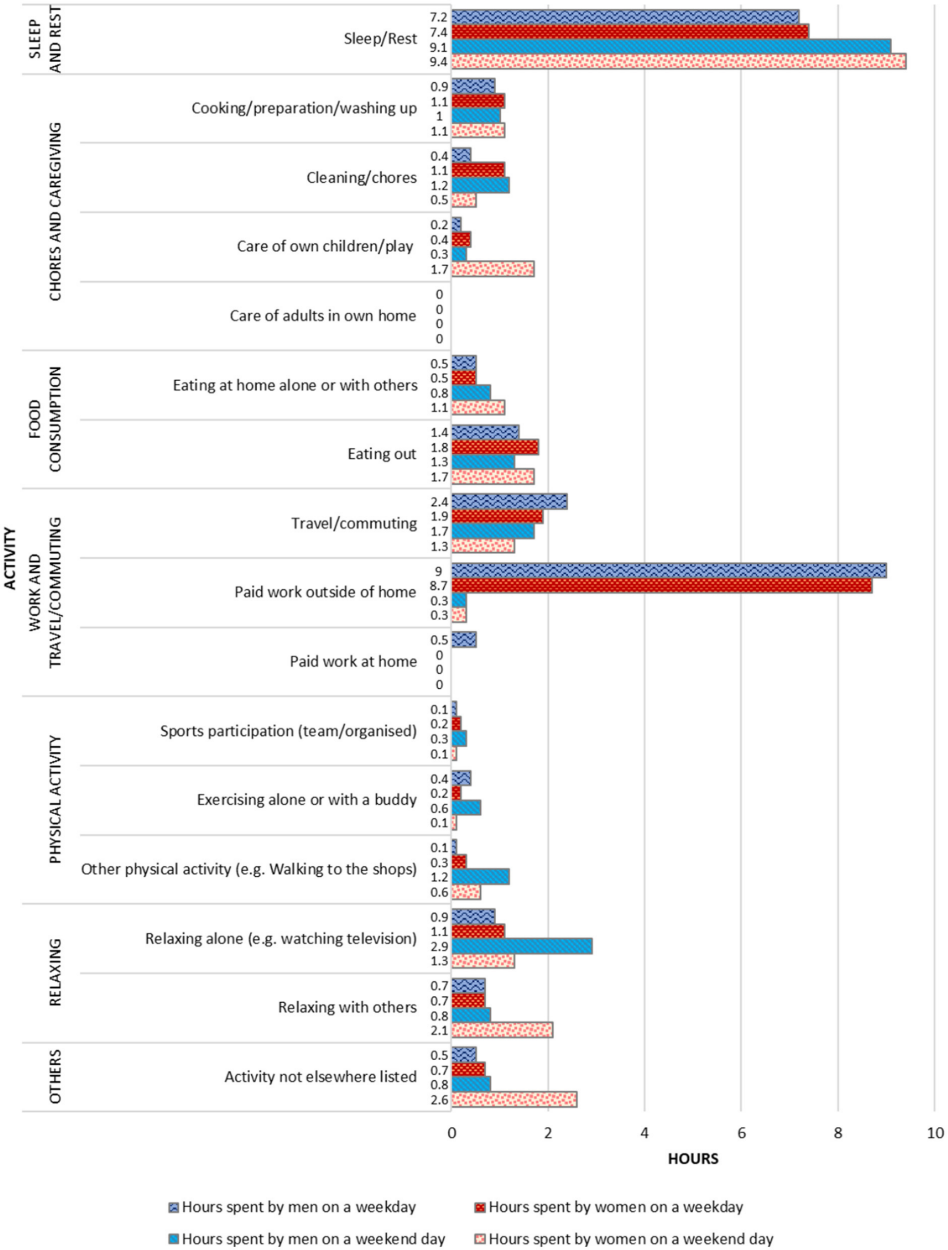
Average hours spent on daily activities by working Singaporean young adults.

Long work hours constituted the greatest time demand on young people during the week. The study participants spent most of their waking hours on weekdays at work (daily average 8.7–9.0 h) and the remainder commuting (1.9–2.4 h) and on food consumption, mainly from food establishments (1.9–2.3 h) (Figure 3). Most participants began work at designated times but often felt compelled to work additional hours at the end of the working day to complete tasks and meet deadlines. Their weekly working hours ranged from 40 to more than 70 h a week, with those in the finance and accounting industry working the longest hours (data not shown). Although the Employment Act 1968 (51, 52) imposes a limit of 44 working h per week, participants worked 47 h per week on average (men 47.3, women 46.7). In total, 20 participants worked on average more than 44 h a week with eight having contracted working hours of between 45 and 50 h.

The Employment Act 1968 (Singapore) (51, 52) mandates only low paid (SGD2600 per month), and manual laborers are paid for overtime work which excludes white-collar professionals and managers. Most participants were, therefore, excluded from overtime payments as a participant employed at a managerial level explained as follows:

*So sometimes I need to do more things, I will stay beyond [normal working hours] … unpaid… I'm not protected in the union agreement [light chuckle] … I'm at the managerial level, so it's not protected*.

*-Lim, male, 33, master's, human resources and statistics analyst, married, SGD 6,000–10,000/month*.

Participants also responded to work-related messages outside of working hours, thereby blurring the boundaries between formal working hours and non-working hours.

As shown in Figure 3, approximately two-thirds of the weekday were taken up by work and sleep.

Even though most participants' work was sedentary, they often reported feeling tired before, during, and after work. Despite sleeping on average 7.3 h each night, many stated that it was normal for them to feel tired all the time. They also felt that they did not have control over the hours they work. To de-stress or unwind from work, they spent their non-work time (approximately 2–4 h per day) commuting, on sedentary entertainment, and sitting with family and friends—often over meals. Most did not do paid work on the weekends, instead relaxing at home or socializing with friends. On average, men and women slept more than 9 h on a weekend day, reinforcing their comments about feeling mentally and physically exhausted. Women spent some additional time with children.

Gendered differences appeared in the allocation of time and work. On average, women did more chores and housework than men on weekdays, and men did more chores and housework than women on weekends, regardless of whether or not they lived in parental homes (Figure 3). Overall, women did more household chores in addition to childcare. In all households with children, participants had help with childcare from family members, live-in domestic helpers, and childcare services so that they could work long hours.

### *Nobody in my office actually leaves at 5 o'clock:* social norms and long work hours

The influence of long work hours and demanding work environments was evident in participants' responses regarding their reasons for their work satisfaction ratings. When participants were asked to elaborate on their ratings, their reasons included working hours, the workplace environment, remuneration, time pressures, allocated work tasks, and feelings toward work tasks. The reported average rating was 5.4, while the mode was 5 (*n* = 14) with a range of 4 to 7 on a scale of 1 (extremely dissatisfied) to 7 (extremely satisfied) (Table 2). There were no observable differences between male and female participants' workplace satisfaction attributes.

**Table 2:** Participants' responses to the time pressure scale question (40) and their work satisfaction rating (39).

	**Total (*N =* 33)**	**Men (*N =* 15)**	**Women (*N =* 18)**
	***N*** **(%), all such values**		
**Response to ‘How often do you feel rushed or pressed for time?'**			
Almost always	6 (18.2)	2 (13.3)	4 (22.2)
Often	7 (21.2)	4 (26.7)	3 (16.7)
Sometimes	14 (42.4)	5 (33.3)	9 (50.0)
Rarely	3 (9.1)	2 (13.3)	1 (5.6)
Never	3 (9.1)	2 (13.3)	1 (5.6)
**Work satisfaction rating**			
1–Extremely dissatisfied	0 (0.0)	0 (0.0)	0 (0.0)
2	0 (0.0)	0 (0.0)	0 (0.0)
3	0 (0.0)	0 (0.0)	0 (0.0)
4	5 (15.2)	2 (13.3)	3 (16.7)
5	14 (42.4)	6 (40.0)	8 (44.4)
6	9 (27.3)	4 (26.7)	5 (27.8)
7–Extremely Satisfied	5 (15.2)	3 (20.0)	2 (11.1)

When asked about their work satisfaction ratings during the interviews, participants consistently spoke about the pressure to meet tight deadlines which left them feeling stressed, particularly during busier seasons such as at the end of the financial year. In response to the time pressure scale (Table 2), 13 participants reported that they were almost always or often rushed or pressed for time at work. These participants worked in banking and accounting, human resources, medical, and marketing, or as managers. It appears that time pressure is not reduced by working longer hours as 12 of them work more than 44 h per week. However, the work satisfaction ratings of the 13 participants who felt they were almost always or often rushed or pressed for time at work ranged between 4 and 6.5, indicating that time pressure at work is not decreasing their job satisfaction ratings. Regardless of their workplace satisfaction ratings, many participants intended to change jobs within the next few years to one that better aligns with their needs for remuneration, training and development, progression, and enjoyment.

Participants revealed multiple reasons for working long hours. They were influenced by senior people who tended to work even longer hours due to added responsibilities and tasks. Sana described how despite one of her bosses negotiating his contract to allow him to end work at 6 p.m., to spend more time with his family, he always ended work after 6 p.m. and would work till much later when required.

*Yeah, and he's really unlucky like sometimes our clients will email us at like 5:30 p.m., “I need this done by tomorrow morning”, so he will stay back late, or he will go home and have dinner, and then you will see his email coming in at like 1 a.m. [sic]*

*- Sana, female, 24, bachelor's, financial public relations, never married, SGD 3,000–4,000/month*.

At 18 years of age, Singaporean men (excluding first-generation permanent residents) are required to enlist for 2 years of full-time national service. Consequently, some male participants felt that the years in National Service were a disadvantage compared to other people, including women of the same age, who may have additional work experience or have completed a master's degree by the time they started work. They felt they needed to work harder to catch up to their peers.

Regardless of their salary, most participants were worried that they did not earn enough to start a family or sustain their family's lifestyle into the future. Unmarried and childless participants worried about the costs of childcare, family expenses, buying a house or a car, and general inflation rates. Unmarried participants usually lived in their parents' homes to save for housing, future expenses, and to accrue general savings, citing rising costs of living. For example, a participant with a housing loan on a flat purchased with siblings explained as follows:

*The lifestyle here is actually quite expensive, yeah, if I'm not wrong, I actually read an article, it's like, a comfortable level is actually about 5,000 [a month] … Yeah, 3 to 4 [thousand a month], not really [enough], because you have to deduct your own monthly expenses, your bills, your housing loan, everything. So, after you deduct, you don't actually have much left*.

*-Mr. Tan, male, 32, diploma, banking, never married, SGD 3,000–4,000/month*.

Securing a well-paid job was an important sociocultural milestone for participants as was being able to provide a stipend for parents. This strong sense of filial piety is characteristic of collectivist societies and has also been fostered in Singapore through the Maintenance of Parents Act (53) which mandates that adults provide financial support for parents of retirement age who are unable to maintain themselves adequately. Many participants provided monthly stipends, usually a set amount or a percentage of their monthly income—further driving the need for higher pay. Regardless of ethnic background, participants commented that they were taught to support their parents from a young age as it was part of their role as children.

*I think we are sort of brought up in a very traditional family, so since young my mum would tell us, hey, I think it's inculcated that she gives money to her mum, my dad does the same, on a monthly basis, and with traditional Chinese families, how much you give a sort of like, although it's not correlated to xiào [*孝*- Chinese for filial]… being filial, but it sort of adds like a tangible number and uh... how did I decide on the amount... uh... close to a thousand but not a thousand, cause parents paid for uni right?*

*- Kenneth, male, 27, bachelor's, banking, never married, SGD 4,000–5,000/month*.

Participants felt they should work hard while they were still young and able to cope with the physical and mental demands. They thought that long hours were required to advance careers and gain better job stability and financial security. Furthermore, they needed to tolerate their work conditions and maintain their job performance to keep their current jobs in what was perceived to be a fiercely competitive job market. To further their careers, some participants also undertook time-demanding training outside of work to improve their future promotion prospects and employability (categorized under “others” in Figure 3). For example, two women, spent a significant amount of time outside work on their counseling diploma and degrees, hoping to change careers or have an alternative career in the future. Another chose to quit his job after working for 1 year to attain a university degree with plans to re-join the workforce for better opportunities and pay due to the fact that degree graduates tend to have higher salaries (54).

### *I'll just eat it [breakfast] on the desk*: the influence of work on eating practices

During interviews, participants described negotiating demands between their workplace, workload and hours worked, and the need to eat. For convenience and to save time, many participants purchased breakfast on the way to work to eat at their workstations. All participants stated that they usually ate out at lunchtime although one participant also bought lunch for her boss because there was no time scheduled into his diary to eat. She explained that his schedule influenced his diet, saying: …* because usually he's in a rush, so usually I don't get uh to buy noodles and soup and stuff*. However, work lunches were viewed as an opportunity to bond with work colleagues and were a brief respite from work. They mainly ate at the ubiquitous food courts and hawker centers within walking distance from their workplaces. It was also common to share unhealthy snack foods at work. However, many participants noted that because of work-driven time pressure, they reduced mealtimes and bought convenient and cheap foods, especially when they were working overtime. Many participants noted that they gained weight once they started work. As Yi Wei described.

*So, when I started working, I was about 65 kg, and the maximum is about 77 kg, and now it's 69… my first job is auditor, so not sure if you know the auditor working hours are quite crazy. So, you work until midnight, so at midnight when you're hungry you can order things like pizza? And McDonald's, so that is the reason why I gained a lot of weight. And when I joined [undisclosed] bank in Malaysia, then I, I get back more work–life balance, then I have more healthy food then that is the reason why I lose my weight until now, I am around 69. [sic]*

–* Yi Wei, male, 30, professional training certificates, operation risk manager, married, SGD 6,000–10,000/month*.

Participants also blamed stress on eating unhealthy foods and snacks.

*When I'm working harder than usual, when deadlines are getting closer yea… Stress eating … Yea, but I do control myself though, like one packet, 2 packets at most … Granola bars, all these, not those like Oreos. [sic]*

*- Mr. G, male, 23, diploma, management consultant, never married, SGD 1,000–2,000/month*.

However, they may also miss meals or eat irregularly due to stress and tight deadlines.

*I don't eat anything…It's like, if you are put on a project, you just don't have time to eat! You don't feel hungry… I might be eating something but it's usually small food… It's more like I have work to finish first*.

*-Mina, female, 24, bachelor's, auditing, never married, SGD 3,000–4,000/month*.

Overall, participants' accounts suggest their work life was a major influence on their eating practices and they were left feeling they had little time and energy for healthy food practices.

### *I would love to exercise every day, but I can't do it:* the influence of work on physical activities

This participant explained that her work schedule did not allow her to exercise even though she wakes fairly early but does not arrive home until 9 p.m. Her experience was not unusual; participants spent most of their waking hours at work (Figure 3), usually in desk-based jobs. On average, participants spent an average of 10 hours (between 4 and 17 hours) a day sitting at a desk, on public transport, or resting after work (Figure 3). Yi Ling, who typically spent 13.5 h a day sitting, largely due to her long hours working as an accountant, commented that she often feels like a “*dead person*”. Articulating the economic imperative to be sedentary another person said that he is “*paid to sit down to do work*”.

The WHO physical activity guidelines recommend per week: 150 min of moderate-intensity aerobic exercise or 75 min of vigorous-intensity aerobic exercise; and muscle strengthening activities on two or more days (55). More than half of the participants (66.7%−12 men and 10 women) took part in recreational physical activity at least once in a typical week. Yet, many participants did not meet the guidelines despite most participants (81.8%−12 men and 15 women) performing moderate or vigorous intensity exercise, largely through incidental activity. During the week, participants spent about 28 min per workday being active, mainly walking to catch public transport and to food establishments.

The main barriers to physical activity were lack of time and lethargy, especially after a long day of work.

*Because I do not really have time to exercise and yes usually after work my work ends about 7:30 to 8 p.m. So, if you go for dinner, it will be from about 8 o'clock to 9 p.m. and you go home, you just shower, and you do not really have time to exercise. Then, Saturday and Sunday usually is spending time at home and don't usually feel like going out, that is the reason why. [sic]*

–* Mr. Tan, male, 32, diploma, banking, never married, SGD 3,000–4,000/month*.

Others explained that their exercise cut into sleep time:

*With work right, it takes up pretty much the whole day, and then sometimes you want some time for yourself, so [exercise] just eats into your sleep, right?*

*-Kenneth, male, 27, bachelor's, banking, never married, SGD 4,000–5,000/month*.

Additionally, some associated seniority with a lack of time for other activities.

*I was standing at the entrance [of the workplace]…looking at all the senior managers and everyone and I'm like “They all look so fat. I'm depressed” and that's what I thought, because what if I become a senior manager? or a manager? So basically they sit there and work all the time*.

*-Mina, female, 24, bachelor's, auditing, never married, SGD 3,000–4,000/month*.

Those that did exercise recognized its benefits.

*In general, I don't feel stressed so easily, I think because I manage it well through exercise, so because of such avenues where I can release my stress, I feel like, no matter how pressurizing my work is, I can manage*.

–* Ali, male, 25, bachelor's, consulting and programming, never married, SGD 4,000–5,000/month*.

Even though Singapore has well-lit streets and facilities offering around the clock opportunities for exercise, many participants said they were too mentally exhausted to do much physical activity, and some did not enjoy it. Instead, they spent their remaining waking hours socializing, watching television, and being online. The mothers in the study had an additional time barrier to exercise because of their caring duties. They preferred to fit exercise in during lunch breaks, as they prioritized childcare outside of working hours. However, some mentioned playing with their children as a form of exercise.

## Discussion

This study focuses on how Singaporean work culture, in particular long working hours, is perceived by young adults (Figure 2). Their accounts demonstrate how their long working hours influenced their diets and physical activity—neither of which conform with health guidelines. Despite the existence of the Employment Act (Singapore Government 1968) capping work hours at 44 h per week, most of our white-collar participants were not protected by the Act and many (20, 60.6%) worked more than 44 h per week without additional remuneration. Full-time Singaporean employees work for an average of 45.4 h per week (54). Other studies have found that for many Singaporean workers (57%), long working hours impact negatively on their work–life balance (56). Participants in this study felt that they did not have control over the duration of working hours, and they subjectively experienced work-related stress and fatigue. They accepted that these conditions due to a strong cultural norm that long work hours were necessary for a successful career, particularly in white-collar and middle management positions (19).

In this regard, these young Singaporean workers conform with other young adults who are transitioning into a new phase in life and are at risk of weight gain due to changes in their lifestyle practices (20). In Australia, which has lower average work hours than Singapore for full-time workers (37.9 hours) (57), long work hours contribute to a lack of time for self-care and health maintenance (11, 12, 58). Although Singapore's long working hours have become normalized, the mental health of working Singaporeans is 13% lower than the general population, and one in six working Singaporeans experienced relatively high levels of stress in comparison to 1 in 10 non-working adults (59).

Singapore's highly accessible food environment makes it possible to eat every meal out with 77.3% of Singaporeans usually having at least one meal outside of the home (60). Participants also ate at least one meal a day outside of the home, especially on weekdays. Commercially prepared food is often less healthy than home-cooked meals due to added fats and sugars and may contribute to weight gain (61, 62). With regards to physical activity, the main reasons for participants' lack of exercise concurred with the findings of a sports participation survey which found that inactivity among those aged 20 to 39 was due to a lack of time due to work (61.0%), no interest in sports (47.0%), and a lack of time due to caring for family (29.0%) (63).

Eating out, particularly with family and friends, sedentary activities, and sleeping for longer were used by participants to mitigate the stress and fatigue from long working hours. Indeed, this study suggests that they are more concerned with managing their emotional and mental wellbeing than their physical health. They appeared to recognize that socializing over meals possibly conferred mental health benefits. Self-care in the form of physical relaxation and psychological detachment from stressors has been shown to be beneficial and acts as a buffer for stress and time pressure (64). However, their accounts suggest that they are at risk of poorer social, mental, and physical health which may lead to the development of burnout syndrome (65) and stress-related eating, which further contributes to overweight and obesity (15–18).

Although this study is not focussed heavily on gendered analysis, we identified some gender differences. Once female participants had children they became time poor due to social expectations to perform most childcare, housework, and the emotional work needed to manage children's schedules, domestic chores, to run the household smoothly, and to maintain relationships. These expectations and practices, in turn, restricted their physical activities and other aspects of daily life. These patterns are reflected globally, where regardless of employment status, women undertake more unpaid work at home than men (66–68). With the percentage of Singaporean dual-income families increasing from 47.1% in 2010 to 53.8% in 2015 (69), married women with children are increasingly required to juggle work and family commitments. Subsequently, gendered roles at home and at work in addition to higher costs of living, long working hours, and career paths are blamed for low birth rates in other urbanized Asian countries such as Japan and Korea (70–72). There seem to be varying levels of awareness in participants' narratives for the need for more gender-equal work and care arrangements for women.

From the structural emphasis on national economic development (73) and the work and sociocultural environment, young working adults feel pressure to prioritize their jobs and work long hours. Our findings are consistent with surveys showing that Singaporean young adults have concerns about the high living costs, providing for their families, and other factors such as employment, job security, and housing (74, 75). Young adults yearn to earn a living that matches their personal and cultural aspirations and rising living costs (76, 77). Thus, it is common for parents aged 35 and 55 to face financial pressures related to simultaneously supporting their growing children and aging parents (78). Particularly household expenditure had increased from SGD3809 in 2007/08 to SGD4906 in 2017/18 (79) and having children was primarily related to increased expenditures of more than SGD 1,000 per month (80). Working long hours confers monetary benefits and helps keep up with inflation rates (81).

A survey of working Singaporeans showed that attaining higher salaries is their top priority, followed by work–life balance, getting along with colleagues, and interesting work in descending order (82). These priorities were reflected in our participants' generally positive work satisfaction ratings. Similar to other findings (83–85), participants planned to change jobs for better opportunities and career paths rather than because they could not tolerate their current job.

### Strengths and limitations

The use of semi-structured qualitative interviews in this study allowed us to understand how and why young adults respond to the work culture in Singapore and what impacts they think it has on their health practices. This study offers deeper and more nuanced insights that generally align well with existing surveys. As is common with qualitative studies, our findings are not widely generalizable. The analysis offers a snapshot of a specific group of people, who are at high risk of weight gain; that is, they are highly motivated, aspirational, well-educated younger white-collar workers mainly of Chinese ethnic background. We expect our findings to be transferable to similar groups in other modern Asian economies. Blue-collar workers have very different work conditions, and an understanding of their work culture on health-promoting practices would be valuable. Because this was a PhD study, it was limited by a tight time frame and budget. The study provides findings that could benefit from further analysis. For example, there are some contradictions in the participants' accounts. While they expressed dissatisfaction about the time constraints of work hours and work culture on being healthy, they accepted the overall logic of Singaporean conditions. In addition, time-use diaries and longitudinal cohort studies of young people would strengthen our knowledge of relationships between sociocultural factors, work, and urban living on health behaviors in Asian settings. To date, time-use surveys have not been widely collected in Singapore (86). While participants were asked about their food choices, empirical data on their actual food intake were not collected which limited the ability of the study to gauge the nutritional quality of participants' diets. In the future, a deeper qualitative analysis of gendered differences and norms could provide useful insights by using a relatively new approach to employment, diet, and health in Singapore.

## Conclusion

To the best of our knowledge, this is the first Singapore-based study investigating how work hours and work culture influences the lives and health of working Singaporean young adults. Participants' narratives show that their long working hours discourage them from health practices in line with existing literature. However, long working hours are perceived as non-negotiable as Singaporean sociocultural norms and institutions support a culture of hard work, and by extension, long working hours to keep up with local and global competition and maintain economic development. Furthermore, Singapore's high costs of living present a significant obstacle for young adults looking to establish themselves financially, provide for their families, and plan for their future. These factors create conditions in which young adults feel obliged to work long hours and maintain a level of work performance to attain financial and job security and stability. While these conditions support Singapore's impressive economic development, long working hours and consequent reduction in health-promoting practices are likely to continue to impose long-term population health implications for Singapore.

## Data availability statement

The datasets presented in this article are not readily available because they contain information that could compromise research participants' privacy and consent but are available from the corresponding author on reasonable request. Requests to access the datasets should be directed to JL, Jodie.Leu@alumni.anu.edu.au.

## Ethics statement

The studies involving human participants were reviewed and approved by the Australian National University Human Research Ethics Committee and the National University of Singapore Institutional Review Board. The patients/participants provided their written informed consent to participate in this study.

## Author contributions

JL conceived and designed the study under the supervision of CB, GS, MK, and SR. JL was involved in the recruitment, data collection, data preparation, analysis, and wrote the original draft. All authors reviewed and approved the final manuscript. All authors discussed the interpretation and presentation of analyzed data.

## Footnotes

^1^As exchange rates have changed over the years since data collection began in 2016, no exchange rate equivalent is provided for Singapore Dollars (SGD).
