# Corrigendum: Hard work, long hours, and Singaporean young adults' health—A qualitative study

**DOI:** 10.3389/fpubh.2024.1409963

**Published:** 2024-05-06

**Authors:** Jodie Leu, Salome A. Rebello, Ginny M. Sargent, Matthew Kelly, Cathy Banwell

**Affiliations:** ^1^National Centre for Epidemiology and Population Health, The Australian National University, Canberra, ACT, Australia; ^2^Saw Swee Hock School of Public Health, National University of Singapore, Singapore, Singapore

**Keywords:** work time, health practices, diet, physical activity, obesity, health promotion, preventing non-communicable diseases, burden of disease

In the published article, there were errors in Table 1 as published. The percentage of Indian male participants in the study was displayed as (33.3). The correct percentage is (20.0). The number of male participants who were of normal weight and corresponding percentage was displayed as 8 (53.3). The correct number and percentage is 10 (66.7). The number of male participants who were overweight and corresponding percentage was displayed as 5 (33.3). The correct number and percentage is 3 (20.0). The number of all participants who were of normal weight and corresponding percentage was displayed as 20 (60.6). The correct number and percentage is 22 (66.7). The number of all participants who were overweight and corresponding percentage was displayed as 7 (21.2). The correct number and percentage is 5 (15.2). The percentage of participants who earned 6000-10,000SGD/month was displayed as (18.1). The correct percentage is (18.2). The corrected Table 1 and its caption ‘Sociodemographic information of participants.' appear below.

**Table 1:** Sociodemographic information of participants.

	**Total (*N* = 33)**	**Men (*N* = 15)**	**Women (*N* = 18)**
***N*** **(%)**		
**Ethnicity**			
Chinese	23 (69.7)	11 (73.3)	12 (66.7)
Malay	6 (18.2)	1 (6.7)	5 (27.8)
Indian	3 (9.1)	3 (20.0)	0 (0.0)
Other	1 (3.0)	0 (0.0)	1 (5.6)
	Mean ± SD		
**Age (years)**	28.7 ± 0.7	29.1 ± 3.2	28.3 ± 4.6
**BMI** (**kg/m**^**2**^)	22.3 ± 3.8	24.6 ± 3.0	20.5 ± 3.6
	N (%)		
**BMI categories**			
Underweight < 18.5	4 (12.1)	0 (0.0)	4 (22.2)
Normal weight, 18.5–24.9	22 (66.7)	10 (66.7)	12 (66.7)
Overweight, 25.0–29.9	5 (15.2)	3 (20.0)	2 (11.1)
Obese ≥ 30	2 (6.1)	2 (13.3)	0 (0.0)
**Living arrangements**			
With family	28 (84.8)	12 (80.0)	16 (88.9)
With housemates	3 (9.1)	2 (13.3)	1 (5.6)
Alone	2 (6.1)	1 (6.7)	1 (5.6)
**Marital status**			
Never Married	23 (69.7)	10 (66.7)	13 (72.2)
Married	10 (30.3)	5 (33.3)	5 (27.8)
**Number of children**			
0	27 (81.8)	13 (86.7)	14 (77.8)
1	1 (3.0)	1 (6.7)	0 (0.0)
2	4 (12.1)	1 (6.7)	3 (16.7)
3	1 (3.0)	0 (0.0)	1 (5.6)
**Highest completed level of education**			
Secondary	1 (3.0)	0 (0.0)	1 (5.6)
Diploma	8 (24.2)	3 (20.0)	5 (27.8)
Bachelor's	20 (60.6)	9 (60.0)	11 (61.1)
Professional certificates	1 (3.0)	1 (6.7)	0 (0.0)
Master's	3 (9.1)	2 (13.3)	1 (5.6)
**Occupation**			
Legislators, senior officials and managers	2 (6.1)	0 (0.0)	2 (11.1)
Professionals	24 (72.7)	13 (86.7)	11 (61.1)
Associate professionals and technicians	1 (3.0)	1 (6.7)	0 (0.0)
Clerical support workers	5 (15.2)	1 (6.7)	4 (22.2)
Service and sales workers	1 (3.0)	0 (0.0)	1 (5.6)
**Monthly income inclusive of Central Provident Funds (SGD)**			
1,000–2,000	2 (6.1)	1 (6.7)	1 (5.6)
2,000–3,000	7 (21.2)	2 (13.3)	5 (27.8)
3,000–4,000	6 (18.2)	2 (13.3)	4 (22.2)
4,000–5,000	12 (36.4)	5 (33.3)	7 (38.9)
6,000–10,000	6 (18.2)	5(33.3)	1 (5.6)

In the published article, there was an error in the numbers reported in relation to the number of participants who met the physical activity recommendations.

A correction has been made to **Results**, “*I would love to exercise every day, but I can't do it: the influence of work on physical activities”*, Paragraph 2. This sentence previously stated:

Nevertheless, more than half of the participants (66.7%−12 men and 10 women) took part in physical activity at least once in a typical week, although fewer (39.4%−7 men and 6 women) met the recommendation of 150 min of moderate-intensity aerobic exercise or at least 75 min of vigorous-intensity aerobic exercise in a week (55).

The corrected sentence appears below:

The WHO physical activity guidelines recommend per week: 150 min of moderate-intensity aerobic exercise or 75 min of vigorous-intensity aerobic exercise; and muscle strengthening activities on two or more days (55). More than half of the participants (66.7%−12 men and 10 women) took part in recreational physical activity at least once in a typical week. Yet, many participants did not meet the guidelines despite most participants (81.8%−12 men and 15 women) performing moderate or vigorous intensity exercise, largely through incidental activity.

The authors apologize for this error and state that this does not change the scientific conclusions of the article in any way. The original article has been updated.

