# Supplementary material for: Hard work, long hours, and Singaporean young adults' health—A qualitative study
Source: Front Public Health. 2023 Jun 12;11:1082581. doi: 10.3389/fpubh.2023.1082581 (PMC10291095; doi:10.3389/fpubh.2023.1082581)
Supplement: Supplementary file 1 [file Table_1.DOCX]

Semi-structured Interview Guide

Theme List

- What a typical work day is like
- What kinds of foods are eaten each day and the thought processes behind food preferences and behaviors
- Time pressure as a factor in work and food choices
- Where foods are eaten and why
- Exercise and health behaviors and perceptions in daily life

Interview Questions

Routines and Activities

- Can you tell me about a typical day? Perhaps using the time use survey you have filled out?
  - Are times flexible?
  - When was the last time you had to work overtime?
  - How do you go to and from work?
  - What is the work situation like?
  - Do you walk around a lot or sit down most of the time?

Eating

- So what do you eat during a typical day? Breakfast, lunch and dinner?
- Where do you eat these meals? How far are you willing to travel?
- How long do you take, do you feel like you’re under pressure?
- Why do you pick the foods you eat?
  - Time, cost etc?
- Any foods you avoid? Any cravings?
- Are there any factors at work that makes you want to eat or snack in particular?
  - Do you snack during work?
- Has food preference changed over time?
- For males: Has national service changed any food preferences?
- Do you eat meals with other people? And do they influence what you eat?
  - E.g. family members and friends
- Any social media impact on the foods you eat?
- Does your mood affect what you eat?
  - Weather?
  - Religion and beliefs?
  - Personal health condition or health conditions of family members/friends?
- How about the weekend?
- Do you know how to cook? Do you cook at all?
- Do your finances affect what you eat and buy?
- How much do you spend a week on groceries?
- How much do you spend a week on food you buy, e.g. restaurants, hawker centres?
- What kinds of foods are eaten at home?
- When do you find time to do groceries?
  - And what kinds of foods are stocked up at home?

Weekends? What about weekends?

- Do you think there are differences between eating in and eating out?
  - E.g. time, money, energy, social implications?

Health Behaviors

- What is considered healthy eating, eating well?
- Has your weight changed since starting work?
- Have you ever been on a diet to lose weight or gain weight? What happened and what do you think is the best way for busy people to lose weight?
- Are there anyways you think you can better your diet? In terms of nutrition, health and enjoyment?
- Are there any work initiatives to promote health? Any examples?
- Do you smoke?
- How often do you drink?

Ending Questions

- Do you have any thoughts on how your workplace policies have a role on your behavior?
  - Health promotions, working hours?
- Any food and eating changes expected in the future?
- How do you think you’ll be eating in 1 year, 5 years, and 10 years?
- Any ideas on what can be done to improve eating healthier?
- And the last part, a small survey on incidental exercise.
